# Exploring parent-child relationships in a Swedish child and adolescent psychiatry - cohort of adolescents with internet gaming disorder

**DOI:** 10.1186/s40359-024-02306-3

**Published:** 2025-01-08

**Authors:** Sabina Kapetanovic, Maiken Due Nielsen, Frida André, Sevtap Gurdal, Emma Claesdotter-Knutsson

**Affiliations:** 1 Department of Behavioural Studies, University West, Trollhättan, SW-416 86 Sweden; 2 Faculty of Medicine, Department of Clinical Sciences, Lund University, Lund, Sweden; 3 Region Skåne, Child and Adolescent Psychiatry, Regional Outpatient Care, Lund University Hospital, Lund, Sweden; 4 Department of Psychology, Stockholm University, Stockholm, Sweden

**Keywords:** Parent-child relationships, Internet gaming disorder, Problem gaming, Child and adolescent psychiatry

## Abstract

**Background:**

While recent studies suggest a high prevalence of Internet gaming disorder (IGD) in child and adolescent psychiatry (CAP) clinics, little is known about the factors contributing to problematic gaming among these patients. Given the well-established role of parenting and parent-child relationships in the development of problem behaviors, this study aimed to explore parent-child relationships within a Swedish cohort of CAP patients with IGD.

**Methods:**

A total of 72 adolescents from CAP clinics in Skane, Sweden, diagnosed with IGD based on DSM-V criteria (73% boys), aged 13 to 18 years were included in the study. The adolescents completed the Game Addiction Scale for Adolescents (GASA) and reported on aspects of parent-child communication, such as parental control and adolescent disclosure and secrecy, and family climate. Adolescents were categorized as engaged, problem or addicted gamers based on core approach. Independent sample t-tests, Pearsons’s correlations, and multivariate regression analyses were used to address the study goals.

**Results:**

Independent sample t-tests revealed that girls showed lower levels of parental knowledge than boys. Bivariate correlation analyses showed that IGD-symptoms were related to lower levels of child disclosure, while multivariate regression analyses revealed that higher IGD-symptoms were predicted by high levels of child secrecy and low child disclosure.

**Conclusion:**

Parent-child relationships, in particular adolescent information management to parents, plays an important role for the level of IGD-symptoms in a clinical sample of adolescents. We suggest that therapeutic interventions for IGD should integrate family-focused strategies, such as parent training programs fostering open communication between parents and their children.

**Supplementary Information:**

The online version contains supplementary material available at 10.1186/s40359-024-02306-3.

## Background

Over the past decade, technology has become an integral part of the daily lives of children and adolescents for learning and recreational activities. One leisure activity that has skyrocketed in popularity among young people is gaming. With the rise of mobile devices and consoles, children and adolescents have unprecedented access to offline and online digital games. An epidemiological study from 2021 revealed that 94% of children in Sweden between ages 8 and 12 played online games daily [1]. This trend has raised concerns among parents and healthcare professionals regarding the potential consequences of pathological gaming on child and adolescent development and mental health [2] and psychosocial development [3].

The inclusion of gaming disorder in the *International Classification of Diseases* (ICD-11) [4] and internet gaming disorder (IGD) as a provisional diagnosis in the *Diagnostic and Statistical Manual of Mental Disorders Fifth Edition* (DSM-5) [5] underscores its recognition as a global mental health challenge, standardizing diagnostic criteria and facilitating research on effective interventions. The idea is that such a recognition not only would legitimize gaming disorder as a mental health condition but also would ensure that researchers, clinicians, and policymakers worldwide operate with a shared definition and diagnostic criteria, promoting consistency in research, diagnosis, and treatment. IGD is defined as a pattern of gaming behavior that leads to significant preoccupation with gaming, prioritizing it over other activities and interests, and causing impairment in various aspects of life [4, 5]. To distinguish high-engagement gamers (i.e., those with a strong interest in gaming) from problem- (i.e., those with impaired control and some harmful effects) and addicted gamers (i.e., those who show signs of addiction with harmful effects) researchers suggest that emphasis should be placed on a smaller number of core symptoms [6]. This “core approach” emphasizes symptoms of withdrawal, relapse, conflict, and consequences as key symptoms of problematic gaming. While the presence of no core criteria would indicate engaged gaming, the presence of two or three core criteria would indicate problem gaming and four core criteria would indicate addictive gaming [6, 7]. In this study, we distinguished adolescents based on core approach criteria to obtain a more clinically relevant and nuanced understanding of how gaming behaviors interact with family dynamics.

The prevalence of problematic gaming is heterogeneous, depending on cultural setting, however in general it is suggested that the prevalence rates across countries is about 3% [8], and most prominent among adolescents and males. Specifically, among Swedish youth, the prevalence rates range from 1 to 2% [9]. On the other hand, among a clinical sample of youth in Swedish child and adolescent psychiatry (CAP) clinics, the number is higher, with 33% of children and adolescents fulfilling the criteria for IGD [10]. In both general and clinical samples, the prevalence of gaming problems is larger in boys than in girls [8, 10]. Several risk factors for IGD have been identified, including male gender, neurodevelopmental disorder such as attention deficit and hyperactivity disorder (ADHD) and autism spectrum disorder (ASD), being 13 years or older, and the presence of mental health conditions such as depression and anxiety [11–13]. While these individual factors contribute to the risk of IGD, the familial context, particularly the quality of parent-child interactions, could also play a critical role. Indeed, given the increasing prevalence of IGD and its strong ties to psychosocial issues, understanding the familial context, particularly parent-child interactions, becomes critical in addressing adolescent gaming problems. As children and adolescents are embedded within the family [14], it is likely that the quality of parent-child relationship would play a significant role for the development of problem behavior and mental illness, including IGD. In the general population of adolescents, high-quality parent-child relationships, characterized by appropriate monitoring, support, and open communication, are associated with positive developmental outcomes [15]. Indeed, adolescents with high-quality relationships with parents and other family members are less likely to develop problem behaviors and unhealthy habits, such as excessive screen use [16, 17].

Positive parenting practices, including rule-setting and parental knowledge of adolescents’ activities have been considered crucial for guiding adolescents toward a healthy developmental path. Traditionally [18], parental knowledge of adolescent activities and whereabouts has been referred to as parents’ active monitoring efforts, such as parents having rules and behavioral expectations for the adolescent (i.e., parental control) and parents’ tracking of adolescent activities through questions and queries (i.e., parental solicitation). More recent theoretical assumptions [19] and empirical research [20] suggest that parental knowledge of adolescent activities is generally obtained through adolescents’ own active information management, including sharing information about one’s whereabouts and activities through open communication (i.e., adolescent disclosure). Moreover, adolescent disclosure, as a proxy for trustful and warm parent-child relationships, is, to a greater extent than parents’ active efforts, associated with lower engagement in risk behaviors [21]. Conversely, withholding information from parents, albeit to some extent normative during adolescence, could be a sign of problems in parent-child interactions [22] which poses threat to adolescent psychosocial functioning [16, 17]. Notably, while parents’ active efforts may indeed reduce the likelihood of adolescents engaging in risk behaviors [16], these same strategies may contribute to adolescents feeling being overly controlled by parents [23]. Adolescents who perceive being overly controlled by their parents report poor parent-child relationships and to some extent poorer psychosocial functioning in comparison with adolescents from non-controlling dynamics [24].

Research on parent-child relationships in children and adolescents with IGD has predominantly been conducted in the general population [25]. Findings from these studies have yielded inconsistent results, with some studies suggesting that problem gaming is associated with higher levels of family conflict, lower levels of family cohesion, and poorer family relationships [25–27], although the effect size for association between family factors and gaming is generally small [28]. Other research suggests there is little to no association between the parent-child relationship and gaming behavior [29]. Indeed, King and Delfabbro [27] investigated features of the parent-child relationship, such as trust and communication, in relation to IGD-symptoms in adolescents. While the results revealed that adolescents with higher IGD-symptoms reported less trust and communication than adolescents without gaming problems, parent-child relationships did not predict the level of IGD-symptoms nor mediate the association between time spent gaming and the level of IGD-symptoms.

How parent-child relationships manifest among children and adolescents within the clinical samples is yet to be understood. Despite the characteristics that differentiate clinical samples of adolescents with IGD, particularly regarding mental health co-occurrence [10], studies on parent-child relationships in relation to gaming in these samples are scarce. To date, there are only a handful of studies that have focused on clinical sample of adolescents in relation to gaming. For example, addressing a number of psychosocial risk factors for IGD in a clinical sample of male adolescent gamers (12–18 years of age) scholars showed that participants (*N* = 31) generally rated their family relationships as negative and high in conflict [30]. Moreover, another study testing internet addiction in a clinical sample of youth suggested that parents of adolescents with internet addiction were perceived as emotionally distant and low in caregiving and monitoring [31]. As these studies only include males with addictive gaming [30] or assess gaming as an aspect of internet addiction [31] it remains unclear how the family functioning manifests in other clinical samples specifically characterized with gaming problems. The knowledge gained from the current study would offer unique insights into the dynamics of parent-child relationships and IGD with the results that would have implications for diagnosis, treatment, and prevention efforts.

Considering the extensive amount of literature demonstrating the impact of family and parental factors on different aspects of adolescent development [15–17] it was considered timely to investigate parent-child relationships in a clinical sample of adolescents with IGD. Moreover, as no previous studies have addressed parenting factors differentiating problematic and addicted gamers based on the core approach [6], we also aimed to examine how parent-child dynamics vary across these groups. This exploration may provide insights into specific parental strategies or relationship patterns that could mitigate the risk of escalation from problematic to addictive gaming behaviors. Sweden’s well-developed CAP healthcare system provides a unique context for such research. It offers access to specialized mental health services for children and young people aged 0–17 years and employs multidisciplinary teams, including psychiatrists, psychologists, social workers, and specialized nurses, ensuring holistic care. Swedish CAP clinics adopt an integrated approach to adolescent mental health, with a strong emphasis on family-centered interventions. Accordingly, the main objective of this study was to explore parent-child relationships, including parent-child communication and family climate, within a Swedish cohort of CAP patients with IGD. Specifically, we ask (1) to what extent different aspects of parent-child relationship, including parent-child communication and family climate, relate to child and adolescent IGD-symptoms, and (2) whether there are any differences in parent-child relationships between girls and boys and between problem and addicted gamers in youth in Swedish CAP clinics. Given the inconsistencies in the literature regarding parenting and adolescent IGD symptoms, particularly in clinical youth samples, we refrained from formulating specific hypotheses for this study. However, considering the high prevalence of IGD-symptoms in adolescents seeking treatment at CAP clinics, understanding parent-child relationships may provide valuable insights into the critical factors affecting this particularly vulnerable youth population. Moreover, findings from this study may aid in developing treatment programs for problematic gaming in adolescents attending CAP clinics.

## Materials and methods

### Participants and procedure

This cross-sectional sub-study explored parent-child relationships in a cohort of patients in Swedish CAP clinics who were included in an RCT for gaming treatment [32]. The recruitment for the RCT occurred between January 1st and December 30th, 2022, across four different CAP clinics in Region Skåne, Sweden. Adolescents between age 13 and 18, during their initial visit to a CAP clinic, underwent screening by a clinician for IGD, utilizing the Game Addiction Scale for Adolescents (GASA) [33]. Those who scored a minimum of 3 on a 5-Likert Scale across the seven items met the cut-off for IGD and were subsequently invited to participate in the study. Additional inclusion criteria were having capacity to provide written informed consent and speaking Swedish since all the scales, as well as the screening forms, were gathered in Swedish. Caregivers’ consents were required for children younger than 15 years of age. Basic demographics including gender, age, housing situations, schooling, and diagnosis, were gathered from the CAP journal. For an in-depth description of the RCT study project setup and procedure, see Kapetanovic et al. [34].

A total of 123 patients met the cut off for IGD. Out of those, one patient was excluded due to incorrect inclusion and 10 patients were excluded due to declining to participate after the first information session [32]. Out of *N* = 112 that entered the RCT, a total of *n* = 81 CAP patients agreed to provide data on parent-child relationships, however nine participants did not complete the questionnaire and were therefore excluded. The questionnaires were filled out by adolescents, without their parents present, after the information session. Consequently, the analytical sample for the current study was *N* = 72 (73.6% male), aged 13 to 18 (*M* = 14.5, *SD* = 1.40). The higher proportion of boys (73.6%) reflects broader trends in gaming behaviour, where males are more likely to engage in gaming and develop IGD symptoms [11–13]. Table 1 shows information on the participants’ gender, age, housing situation, and other diagnosis obtained from the patient registry data.

**Table 1:** Sample characteristics

	Frequency	Percent
Total sample	72	100
Gender		
Male	53	73.6
Female	19	26.4
Age, years		
13–15	56	77.8
16–18	16	22.2
Housing situation		
Cohabiting parents	42	58.3
Separated parents	30	41.7
Diagnosis		
ADHD	32	44.4
ASD	10	13.9
Depression	5	6.9
Other diagnosis*	25	34.8
Gaming categories		
Engaged gamer	4	5.6
Problem gamer	43	59.7
Addicted gamer	25	34.7

Note: ADHD Attention Deficit Hyperactive Disorder, ASD Autism Spectrum Disorder; *includes *anxiety disorders* (anxiety disorder, unspecified, ‘mixed anxiety, and depressive disorder, generalized anxiety disorder), *other symptoms and signs involving emotional state*,* obsessive compulsive disorder*,* adjustment disorder*,* pathological gambling*,* and diagnoses primarily used during the psychiatric evaluation phase* (observation for suspected mental and behavioral disorders, general psychiatric examination, not elsewhere classified, examination and observation for unspecified reason, observation following alleged rape or seduction, examination and observation for unspecified reason). All adolescents were enrolled in school at the time of the study

### Measures

#### Internet gaming disorder

The 7-item Game Addiction Scale for Adolescents (GASA) [33] is a self-report measure that assesses adolescents’ gaming problems during the past six months. The scale is frequently used to assess IGD-symptoms among children and adolescents [7] and is based on seven of the nine DSM-5 criteria for problem gaming (salience, tolerance, relapse, mood modification, withdrawal, conflicts, and problems). Items (e.g., “How often during the past six months did you think about playing a game all day long?”) were answered on a 5-point Likert scale, ranging from 1 = *never* to 5 = *very often*. The scale yields two outcome measures: (A) a gaming category (engaged, problem, or addicted) according to the core approach [6], and (B) a continuous IGD-symptoms score with a minimum score of 7 and a maximum score of 35, with higher scores indicating a higher level of IGD symptoms. As suggested by Core approach criterion [6], the respondents who endorsed four or more GASA core criteria (relapse, withdrawal, conflicts, and problems) were categorized as “addicted gamers” (*n* = 25). Individuals meeting two to three of the core criteria were categorized as problem gamers (*n* = 43), and those that endorsed the peripheral criteria but not more than one of the core criteria were categorized as “engaged gamers” (*n* = 4). Cronbach’s alpha for the continuous IGD-symptoms was α = 0.63.

### Parent-child communication

The study employed a somewhat modified version of the scales developed by Kerr and Stattin [19], frequently used among Swedish children and adolescents [23], to assess parent-child communication among children and adolescents. To the original scale including 20 items, we added six items that assessed parent-child communication relating to gaming (see Supplementary Table 1). In total, the questionnaire contained 26 items, covering six subscales. Parental knowledge (α = 0.70) assessed how much parents knew about their children’s whereabouts and activities using six items (e.g., “Do your parents know what you do during your free time?”). Parental solicitation (α = 0.66) assessed the parents’ efforts to gain knowledge of children’s whereabouts and activities using five items (e.g., “Do your parents usually ask you to talk about things that happened during your free time?”). Child disclosure (α = 0.49) was assessed with four items (e.g., “Do you talk at home about how you are doing in the different subjects in school?”). Here, caution should be exercised due to the low reliability of the measure. Child secrecy (α = 0.63) was assessed with three items (e.g., “Do you keep a lot of secrets from your parents about what you do during your free time?”). Parental control (α = 0.73) was assessed using five items (e.g., “How often do your parents set rules or limits on what you do on the internet?”). Finally, child feeling overly controlled by parents (α = 0.79) was assessed with three items (e.g., “Do you feel that your parents demand to know everything?”). Items were answered using a 5-point scale with answer options tailored to fit the items.

### Family climate

The general family climate [35] scale, developed and used among Swedish children and adolescents [16], was used to assess family cohesion and conflict. Participants were asked to assess how well each statement applied to their family on a 4-point Likert scale, ranging from 1 = *not true at all* to 4 = *very true*. Family cohesion (α = 0.80) was assessed with six statements (e.g., “In my family, we help and support one another”), and family conflict (α = 0.63) was assessed with five items (e.g., “Sometimes family members hit each other”).

### Ethics

The study was reviewed and approved by the Swedish Ethical Review Authority (Ref 2019–04797, December 13, 2019). Subsequent amendments have been approved (Ref 2021-05592-01, January 3 202; Ref 2022-01289-02, March 15, 2022). The study procedures were carried out in accordance with the Declaration of Helsinki. All participants were informed that their data would be anonymous and unidentifiable. Informed consent was obtained from the participants prior to partaking in the study. Caregivers’ consent was required for children younger than 15 years.

### Data analysis

Given low interreliability scores in some of the variables (e.g., GASA, child disclosure) we conducted a series of exploratory factor analyses (EFA) to investigate the factors and the loadings of the factors (see Supplementary Table 2). Based on the results from EFA all measures were used in the following analyses. Missing analysis showed that there was not more than 5% internal missingness in the variables of interest, which is why data from all included cases was kept for further analyses.

The mean scores of scales assessing parent-child communication and family climate were used in a series of independent sample *t*-tests for comparison between boys and girls on the one hand and of addicted and problem gamers on the other hand. The selective application of the Bonferroni correction helped balance the risk of Type I errors with the study’s exploratory nature. Hedge’s G was used to calculate the effect sizes given its robustness in small samples [36]. Due to the small number of engaged gamers in the sample (*n* = 4), we excluded this group from comparison analyses. We conducted Pearson’s correlation analyses to investigate the bivariate relations between the level of IGD-symptoms and parent-child relationship and multivariate linear regression analyses to investigate to what extent parent-child relationships predicted IGD-symptoms. All data preparation and statistical analyses were performed in SPSS (IBM SPSS statistics version 27) and the significance level was set to *p* < .05.

## Results

Table 2 shows means and standard deviations of parent-child communication and family climate. Specifically, while parental knowledge and family cohesion are generally high, child secrecy and family conflict are generally low, indicated by the high and low median and mean, respectively.

**Table 2:** Descriptive statistics of parent-child communication and family climate

	*N*	Minimum	Maximum	Mean	Median	Std. deviation
Parental knowledge	72	2.20	5.00	4.32	4.40	0.59
Parental solicitation	72	1.67	5.00	3.52	3.58	0.77
Child disclosure	72	1.25	5.00	3.21	3.25	0.82
Child secrecy	72	1.00	4.67	1.99	1.67	0.87
Parental control	72	1.00	5.00	2.80	2.80	0.88
Overly controlled	71	1.00	5.00	2.77	2.67	1.21
Family cohesion	71	1.80	4.00	3.22	3.33	0.56
Family conflict	71	1.20	3.75	2.14	3.00	0.55

Moreover, boys and girls scored similarly on most scales, however *t*-test analyses showed differences in the mean levels of parental knowledge and child secrecy between boys and girls. While girls (*M* = 3.93 *SD* = 0.75) showed lower levels of parental knowledge (*t* (70) = -3.64, *p* < .001) than boys (*M* = 4.46 *SD* = 0.46), boys (*M* = 1.84 *SD* = 0.70) showed lower levels of child secrecy (*t* (70) = 1.49, *p* = .019) than girls (*M* = 2.39 *SD* = 1.17). However, upon applying the Bonferroni correction to safeguard against Type I error, we found that only parental knowledge reached the threshold of significance (see Supplementary Table 3). Moreover, we also tested whether there were any significant differences in parent-child communication and family climate between the problem and addicted gamers; however, we discovered no significant differences between these groups (see Supplementary Table 4). The absence of differences in parent-child communication between problem and addicted gamers suggests that these dynamics are consistent across IGD severity levels.

Next, we tested bivariate correlations between gaming IGD-symptoms and aspects of parent-child relationships. As shown in Table 3, the results indicated that only correlations between child disclosure and IGD-symptoms were significant, revealing that lower levels of child disclosure were related to lower IGD-symptoms.

**Table 3:** Bivariate correlations between IGD-symptoms and parent-child communication and family climate

Variable	1	2	3	4	5	6	7	8
1. IGD-symptoms	-							
2. Parental knowledge	0.055	-						
3. Parental solicitation	− 0.025	0.196	-					
4. Child disclosure	− 0.240*	0.022	0.511**	-				
5. Child secrecy	0.200	− 0.617**	− 0.076	− 0.066	-			
6. Parental control	0.095	− 0.014	0.339**	0.187	0.245**	-		
7. Overly controlled	0.037	0.170	0.228	− 0.095	0.142	0.375**	-	
8. Family cohesion	0.004	0.415**	0.233	0.327**	− 0.507**	0.031	− 0.107	-
9. Family conflict	− 0.063	− 0.098	0.000	− 0.085	0.147	0.183	0.482**	− 0.390**

Note: * *p* < .05 ** *p* < .001; significant results are in bold

Finally, as shown in Table 4, multivariate regression analysis with all parenting variables of interest, showed that higher levels of child secrecy (β = 0.471 *p* = .009) and lower levels of child disclosure (β = − 0.288 *p* = .049) predicted higher level of IGD-symptoms (*F* [8] = 1.59 *p* = .15, *R*² = 0.17, *R*²*Adjusted* = 0.06).

**Table 4:** Regression analysis with parenting variables predicting IGD-symptoms

Variable	B	95% CI	β	t	*p*
Parental knowledge	1.91	-0.26, 4.05	0.290	1.76	0.084
Parental solicitation	0.57	-0.96, 2.10	0.109	0.74	0.460
Child disclosure	-1.40	-2.80, -0.04	− 0.288	-2.01	**0.049**
Child secrecy	2.16	0.55, 3.76	0.471	2.69	**0.009**
Parental control	0.21	-0.99, 1.42	0.048	0.34	0.732
Overly controlled	-0.43	-1.47, 0.61	− 0.129	-0.83	0.411
Family cohesion	1.27	-099, 3.52	0.175	1.12	0.266
Family conflict	− 0.015	-2.27, 2.24	− 0.002	-0.13	0.990

Note. *N* = 72; CI = Confidence interval for *B; p*-values in bold are statistically significant

## Discussion

As video games and gaming culture continue to evolve with the rapid progression of technology, there is a growing interest in understanding the factors that contribute to problem gaming in children and adolescents [3]. High quality parent-child relationships are generally considered a critical factor for adolescent development [15], which is why it is possible that parent-child relationships are important correlates and predictors of adolescent problem gaming. In this study we explored parent-child relationships, including parent-child communication and family climate, within a Swedish cohort of CAP adolescents with IGD and addressed the potential differences in parent-child relationships among boys and girls, and among adolescents of different severity of IGD (thus problematic and addicted gaming).

Our results show that adolescents within Swedish CAP clinics diagnosed with IGD, in line with the general population of Swedish adolescents [16, 37], yet in contrast to a clinical sample of Spanish male adolescents with IGD [29], report fairly positive parent-child relationships characterized by positive parenting practices and a sense of family cohesiveness. In general, Sweden is a country with parenting norms shaped by a mix of individualism and progressive social norms fostering mutual respect between parents and their children [38]. Such norms contribute to a supportive, respectful, and balanced family dynamic which inevitably have impact on parent-child relationships [21], both in general samples as well as the clinical samples of children. Moreover, parents of children and adolescents with mental health problems are considered highly engaged and involved in their children’s wellbeing and treatment [39] which also may explain the high mean values of adolescents’ ratings of parent-child relationships. On the other hand, it is also possible that these adolescents overrate their parent-child relationships. As suggested by scholars [40], children with neuropsychiatric disorders such as ADHD tend to overestimate the strength of their social relationships - a phenomenon known as the positive illusory bias. As a substantial proportion of the participants (37%) were diagnosed with ADHD this could be the case.

Further, exploring the associations between parent-child relationships and IGD-symptoms we found that lower levels of child disclosure and higher levels of child secrecy were predictive of higher IGD-symptoms in adolescents in our study. This means that adolescents diagnosed with IGD who do not talk to their parents about their activities, or rather withhold information from their parents, including gaming behaviors, show higher IGD-symptoms, and vice versa. These results are particularly compelling, as earlier research with general population of adolescents consistently shows that child disclosure is the major protective factor while child secrecy is the major risk factor for adolescent psychosocial development [16, 19, 23]. The fact that we observe similar patterns in a clinical sample of adolescents with IGD suggests that the dynamics of child-parent communication extend beyond general developmental outcomes to specific behavioral issues such as gaming problems. As parents obtain much of the information of adolescents’ whereabouts and activities from child voluntary disclosure [20, 23], it is possible that adolescents who talk to their parents would be more likely to let their parents be involved in their lives. As such, the adolescents would be more willing to accept the support and guidance from their parents, which in turn could help them with their gaming problems. On the other hand, when adolescents conceal information about their activities, such as gaming, from their parents, it may render them less accessible to parental influence [16]. Although there are different reasons to why adolescents keep secrets from their parents [19], hiding information from parents could also be a sign of poor parent-child relationship quality [22]. These findings reinforce the universality of child disclosure and secrecy as critical aspects of adolescent development, irrespective of whether the adolescents are from clinical or non-clinical settings. It underscores the notion that fostering open communication between adolescents and parents is crucial not only for general well-being but also for mitigating specific behavioral challenges like IGD. These findings have clear implications for clinical practice, particularly in designing and implementing interventions focusing on equipping parents with the skills to foster open, non-judgmental communication with their adolescents, which may encourage greater disclosure and reduce secrecy. For instance, training programs might include modules on active listening and fostering trust, encouraging adolescents to share their gaming habits [41] as such interventions have proven effective in treating adolescents with gaming problems [42].

In the present study, family cohesion, serving as an indicator of relationship quality, did not emerge as a significant direct predictor of IGD-symptoms. Nonetheless, there is a possibility that family cohesion might act as a moderator [16] influencing the effects of other parenting variables and offering additional insights into the findings observed in this study. These ideas need to be explored further, preferably with larger samples and several assessments over time. Then again, caution should be exercised due to the poor goodness of fit (*R*² statistics) observed in the multivariate regression model, which may suggest that other significant predictors of IGD symptoms are not accounted for in the model. Given that children and adolescents attending CAP clinics typically exhibit poor mental health or other emotional symptoms [7], we propose that future studies incorporate patient personal characteristics alongside environmental factors, such as peer and school factors, to enhance understanding of gaming problems in adolescents.

Given that gaming habits and problems differ between boys and girls, we also explored to what extent parent-child relationships differed between genders. Contrary to earlier literature [23], we found that boys reported higher levels of parental knowledge than girls. Given the medium to large effect sizes, we found this result surprising. Indeed, parents are generally more knowledgeable of girls’ activities, not the least as girls tend to share more information with their parents than boys [43]. However, the extent to which parents are informed about their children’s activities is largely contingent on whether adolescents perceive their parents as having the right to know [44]. In this case, it is possible that cultural as well as societal norms relating to gaming may offer some explanation to these results. Although both boys and girls engage in gaming, it is typically considered a male-dominated activity [1], and more socially acceptable for males than females. This may potentially foster more shame and, thus, more secrecy in girls who engage in gaming [45, 46]. Conversely, boys may feel more comfortable sharing their gaming habits and exert less effort hiding their activities from their parents, which is why parents would also have more knowledge of their activities [46]. Interview studies with parents and their adolescent children could offer more explanation to these ideas.

Additionally, we explored potential differences in parent-child relationships between adolescents classified as problematic versus addicted gamers based on the core approach criteria [6]. We found no significant differences between these groups across the parenting variables examined. Even if parent-child relationships and family interactions are important factors in the development of problematic gaming [28], their significance may not necessarily differ between adolescents with problematic and addictive gaming behaviors. It is possible that the role of these relationships reaches a threshold of influence that remains consistent across the two groups. Additionally, the lack of significant results could also be attributed to the relatively small sample size, which may limit statistical power and the ability to detect subtle differences between the groups. Future studies with larger samples are needed to better understand the potential variations in the family dynamics across adolescents classified as problematic and addicted gamers.

### Limitations

This study has some limitations that should be considered when interpreting the findings. The first is the small size of our sample (*N* = 72). Low statistical power because of small sample sizes may pose the risk of producing false negatives or spurious results [47]. This means that differences, or lack thereof, could be the result of the limited sample size rather than actual differences. Our sample was predominantly male (73.6%), reflecting known trends in IGD prevalence [9, 13]. However, this limits the generalizability of findings to female adolescents, who may experience IGD differently or exhibit distinct parent-child relationship patterns. Future research should aim to recruit more participants, particularly girls, who were not well-represented in the present study (*n* = 19). In addition, the low alpha scores for some of the measures (i.e., child disclosure) may also pose threat for the validity and reliability of the measures, and consequently, the study’s findings. Although there may be a number of reasons for low alpha scores, including small sample size and low number of items in the measure, it is also possible that some adolescents included in our study, given the complexity of their clinical situation had difficulties to reflect about their current situation, such as to what extent their gaming behavior had impact on their everyday lives (item 7 in GASA). Notably, such consequences are more likely to be noted by other significant people in children’s immediate environment (e.g., parents or teachers). After conducting EFA: s to understand and secure the structure of the measures, we decided to include all the scales in the analyses. Nonetheless, more research on parent-child communication in clinical samples of adolescents is called for, along with greater attention to the operationalization of both general and specific aspects of parent-child communication for these specific samples. Additionally, although Stattin and Kerr’s [19] parent-child communication scales are widely used in parenting literature [21, 23], we recommend updating these scales to more accurately reflect the contemporary realities of youth, which encompass online activities such as gaming and social media use.

Furthermore, it is noteworthy that participants in our sample generally reported positive parent-child relationships. This positive bias in responses could potentially impact the study results and may have implications for future research, especially when dealing with clinical samples of adolescents. Additionally, caution is advised when interpreting the results, given that they rely solely on self-reported measures from adolescents. Positive bias in self-reports, could inflate ratings of parent-child relationships, warranting the inclusion of multi-informant perspectives in future studies. Since there tends to be a discrepancy between parents’ and adolescents’ reports on health and parenting [48], future studies should contemplate including perspectives from different family members, encompassing both adolescents and their parents. This approach can enhance reliability and mitigate the risk of common method variance. Additionally, our study did not differentiate between adolescents’ relationships with their mothers and fathers. This could have influenced the results, especially considering that more than a third of the participants had separated parents. Differing rules and expectations in separated households, especially when adolescents split their time between them, may impact both their behavior and their relationships with their parents. It is worth noting that parent-child relationships concerning problematic gaming may vary between mothers and fathers [49]. To achieve a more comprehensive understanding of parent-child relationships in the context of problematic gaming within a clinical sample, it is advisable to explore the dynamics of relationships between different parent-child dyads. Finally, given the small sample in our study, we were unable to test whether the associations between IGD-symptoms and aspects of parent-child relationships could be moderated by individual characteristics such as gender. Since both gaming behaviors and parent-child relationships can vary by adolescent gender [1, 20], we recommend that future research with sufficiently larger samples investigate this possibility to provide a more comprehensive understanding of dynamics in parenting and adolescent gaming behaviors.

## Conclusions

Problematic gaming is a rapidly growing area of research, with limited exploration of parent-child relationships in connection to gaming, especially among clinical samples of adolescents with Internet Gaming Disorder (IGD). The present study explored parent-child relationships in a CAP cohort of adolescents diagnosed with IGD. The results indicate that (a) parent-child relationships are generally positive among adolescents in CAP clinics diagnosed with IGD, (b) boys report higher parental knowledge of adolescent whereabouts and (c) child secrecy and child disclosure of everyday activities to parents are important correlates and predictors of IGD-symptoms. Larger and more diverse samples are however needed to validate these findings and to explore gender-specific interventions that can more effectively support adolescents in overcoming gaming-related issues.

Nonetheless, these findings suggest that it may be beneficial to take into account parent-child relationships in treatment and intervention programs targeting gaming issues in CAP clinics. Incorporating parent-child relationship assessments into CAP clinics may enhance the efficacy of interventions for IGD. Specifically, interventions should focus on enhancing child disclosure and reducing secrecy through structured family communication therapies that include both the adolescent and their parents(s), particularly in clinical settings. As gaming problems are a multifaceted issue, interdisciplinary research drawing on expertise from psychology, family therapy, and addiction studies can provide a more comprehensive understanding of the factors that contribute to IGD and its impact on adolescents’ development. Collaboration across disciplines will allow for the design of multifaceted interventions that address not only the gaming behavior itself but also the relational and psychological factors that may exacerbate or alleviate it.

## Electronic supplementary material

Below is the link to the electronic supplementary material.

Supplementary Material 1

Supplementary Material 2

Supplementary Material 3

Supplementary Material 4

## Data Availability

The datasets generated and/or analyzed during the current study are not publicly available because this study’s ethical review does not allow for study data to be in a public repository. The datasets used and/or analyzed during the current study are available from the corresponding author on reasonable request.

## Abbreviations

IGD: Internet gaming disorder
CAP: Child and adolescent psychiatry
DSM-V: Diagnostic and statistical manual of mental disorders, fifth edition
GASA: Game Addiction scale for adolescents
RCT: Randomized controlled trial
ICD-11: International classification of diseases, 11th Revision
ADHD: Attention deficit hyperactive disorder
ASD: Autism spectrum disorder
SPSS: Statistical package for the social sciences
SD: Standard deviation
CI: Confidence interval
EFA: Exploratory factor analyses
