# Supplementary material for: Exploring parent-child relationships in a Swedish child and adolescent psychiatry - cohort of adolescents with internet gaming disorder
Source: BMC Psychol. 2025 Jan 8;13:18. doi: 10.1186/s40359-024-02306-3 (PMC11708115; doi:10.1186/s40359-024-02306-3)
Supplement: Supplementary file 1 — Supplementary Material 1 [file 40359_2024_2306_MOESM1_ESM.docx]

Supplementary Table 1. Overview of the Items in Parent-Child Communication Scale and Parental Rules Scale

| Sub-scale | Items |
| --- | --- |
| Parental Knowledge | Do your parents usually know when you have an exam at school?  In the last month, have your parents ever had no idea of where you were at night?  Do your parents know what you do during your free time?  Do your parents know what you spend your money on?  *Do your parents know how much of your time is spent on gaming?* |
| Parent Solicitation | How often do your parents talk to your friends when they come over to your house?  Do your parents usually ask you to tell them about your friends (for example what they like doing and how they are doing at school)?  During the past month, how often have your parents initiated a conversation with you about your free time?  How often do your parents ask you to sit and tell them what happened at school on a regular school day?  How often do your parents ask you about where you have been after school and what you have done?  *How often do your parents inquire about your gaming activities?* |
| Adolescent Disclosure | Do you usually tell how school was when you get home (what you did, your relationships with teachers, etc.)  Do you talk at home about how you are doing in the different subjects in school?  Do you like to tell your parents where you went and what you did during the evening?  *Do you talk to your parents about your gaming activities?* |
| Adolescent Secrecy | Do you keep a lot of secrets from your parents about what you do during your free time?  Do you hide a lot from your parents about what you do during nights and weekends?  *Do you withhold information regarding your gaming activities from your parents?* |
| Parental Control | How often do your parents set rules or limits on who you spend time with?  How often do your parents set rules or limits on what you do on the Internet?  How often do your parents set rules or limits on how you spend your money How often do your parents set rules or limits on where you go right after school?  *How often do your parents set rules or limits on your gaming activities?* |
| Adolescent Feelings of Being Overly Controlled | Do you think that your parents decide everything in your life?  Does it feel as if your parents demand to know everything?  *Do you feel that your parents are overly engaged in your gaming activities?* |

*Note:* For the purpose of the study, the italicized items are included to capture parent-child communication and parental awareness of their child's gaming activities.
