# Supplementary material for: Exploring parent-child relationships in a Swedish child and adolescent psychiatry - cohort of adolescents with internet gaming disorder
Source: BMC Psychol. 2025 Jan 8;13:18. doi: 10.1186/s40359-024-02306-3 (PMC11708115; doi:10.1186/s40359-024-02306-3)
Supplement: Supplementary file 2 — Supplementary Material 2 [file 40359_2024_2306_MOESM2_ESM.docx]

Supplementary Table 2. Exploratory Factor Analysis of Variables in the Study

| Scale | Item | One-factor solution | % of variance | Two-factor solution | % of variance |
| --- | --- | --- | --- | --- | --- |
| GASA |  |  | 30.95 % |  | 19.65% |
|  | Thinking of gaming | .57 |  | -.33 |  |
|  | Spending more time on gaming | .62 |  | -.29 |  |
|  | Gaming to escape the real life | .33 |  | .72 |  |
|  | Failed to quit gaming | .72 |  | -.06 |  |
|  | Felt bad when not being able to game | .56 |  | .19 |  |
|  | Fighting with others because of gaming | .70 |  | -.08 |  |
|  | Ignored other activities because of gaming | .15 |  | .78 |  |
| Parental knowledge |  |  | 49.77 % |  | 20.19 % |
|  | Having exam | .82 |  | .07 |  |
|  | Where at night | .27 |  | .92 |  |
|  | Doing during free time | .79 |  | -.09 |  |
|  | Spending money | .69 |  | -.38 |  |
|  | Time on gaming | .79 |  | .04 |  |
| Parental solicitation |  |  | 37.91 % |  | 17.99 % |
|  | Parents talk to friends | .54 |  | -.61 |  |
|  | Ask about friends | .63 |  | -.10 |  |
|  | Ask about free time | .75 |  | .24 |  |
|  | Ask about school | .62 |  | .10 |  |
|  | Ask about time after school | .70 |  | -.21 |  |
|  | Ask about gaming activities | .39 |  | .76 |  |
| Child disclosure |  |  | 39.74 % |  | n/a |
|  | Telling parents about school | .54 |  |  |  |
|  | Telling parents about subjects in school | .51 |  |  |  |
|  | Telling parents about activities in the evening | .72 |  |  |  |
|  | Talking to parents about gaming | .72 |  |  |  |
| Child secrecy |  |  | 59.11 % |  | n/a |
|  | Keeping secrets about free time | .73 |  |  |  |
|  | Keeping secrets about nights/weekends | .86 |  |  |  |
|  | Keeping secrets about gaming | .71 |  |  |  |
| Parental control |  |  | 49.51 % |  | 20.11 % |
|  | Rules about friends | .80 |  | -.25 |  |
|  | Rules about Internet | .75 |  | .27 |  |
|  | Rules about money | .81 |  | -.12 |  |
|  | Rules about going out | .69 |  | -.31 |  |
|  | Rules about gaming | .36 |  | .87 |  |
| FOH |  |  | 70.93 % |  | n/a |
|  | Parents decide everything | .88 |  |  |  |
|  | Parents demand to know everything | .88 |  |  |  |
|  | Parents overly engaged in gaming | .76 |  |  |  |
| Family cohesion |  |  | 52.45 % |  | n/a |
|  | Family support | .67 |  |  |  |
|  | Family having fun | .80 |  |  |  |
|  | Getting along | .82 |  |  |  |
|  | Sense of unity in family | .77 |  |  |  |
|  | Do a lot together | .48 |  |  |  |
|  | Do not avoid one another | .75 |  |  |  |
| Family conflict |  |  | 45.05 % |  | n/a |
|  | We fight a lot | .77 |  |  |  |
|  | Hit one another | .70 |  |  |  |
|  | Lose temper | .76 |  |  |  |
|  | Criticize one another | .42 |  |  |  |
|  | Throwing things when angry | .64 |  |  |  |

Note: For some scales, the results showed both one-factor and two-factor solutions. When two-factor solution was present, the additional percentage of variance and factor loadings of the second factor are presented in the table; FOH = Feelings of being overly controlled; n/a = not applicable
