# Supplementary material for: Exploring parent-child relationships in a Swedish child and adolescent psychiatry - cohort of adolescents with internet gaming disorder
Source: BMC Psychol. 2025 Jan 8;13:18. doi: 10.1186/s40359-024-02306-3 (PMC11708115; doi:10.1186/s40359-024-02306-3)
Supplement: Supplementary file 3 — Supplementary Material 3 [file 40359_2024_2306_MOESM3_ESM.docx]

**Supplementary Table 3.**  *Independent Sample t-Test Showing Mean Differences between Boys and Girls in Terms of Parent-Child Communication and Family Climate*

|  | Valid N | Mean (**SD**) ♂ | Mean (**SD**) ♀ | *t*-test (*df*) | *p*-value | Hedge’s *G* |
| --- | --- | --- | --- | --- | --- | --- |
| Parental knowledge | 53♂, 19♀ | 4.46 (0.46) | 3.93 (0.75) | -3.64 (70) | **.001** | 0.96 |
| Parental solicitation | 53♂, 19♀ | 3.53 (0.73) | 3.50 (0.88) | -0.14 (70) | .886 | 0.04 |
| Child disclosure | 53♂, 19♀ | 3.12 (0.77) | 3.46 (1.03) | 1.49 (70) | .141 | 0.39 |
| Child secrecy | 53♂, 19♀ | 1.84 (0.70) | 2.39 (1.17) | 2.39 (70) | .019***** | 0.63 |
| Parental control | 53♂, 19♀ | 2.84 (0.87) | 2.71 (1.05) | -0.51 (70) | .612 | 0.13 |
| Overly controlled | 52♂, 19♀ | 2.88 (1.19) | 2.44 (1.25) | -1.29 (69) | .200 | 0.34 |
| Family cohesion | 52♂, 18♀ | 3.27 (0.50) | 3.11 (0.67) | -1.07 (68) | .287 | 0.29 |
| Family conflict | 52♂, 19♀ | 2.10 (0.56) | 2.25 (0.54) | 1.01 (69) | .316 | 0.27 |

Note: Hedge’s *G* < 0.2 small effect, < 0.5 medium effect, < 0.8 large effect *Did not hold for Bonferroni correction; *p*-value in bold is statistically significant
