# Supplementary material for: Exploring parent-child relationships in a Swedish child and adolescent psychiatry - cohort of adolescents with internet gaming disorder
Source: BMC Psychol. 2025 Jan 8;13:18. doi: 10.1186/s40359-024-02306-3 (PMC11708115; doi:10.1186/s40359-024-02306-3)
Supplement: Supplementary file 4 — Supplementary Material 4 [file 40359_2024_2306_MOESM4_ESM.docx]

**Supplementary Table 4.** Independent Sample t-test Showing Mean Differences between Problem and Addicted Gamers

|  | Problematic | |  | Addicted | | *t-*test (df) | P-value | 95% CI of Mean Difference | |
| --- | --- | --- | --- | --- | --- | --- | --- | --- | --- |
|  | *N* | *M* (*SD*) |  | *N* | *M* (*SD*) |  |  |  |  |
| Parental knowledge | 43 | 21.39 (3.15) |  | 25 | 22.00 (2.91) | -0.783 (66) | 0.436 | -2.15, 0.94 |  |
| Parental solicitation | 43 | 21.56 (4.33) |  | 25 | 19.68 (4.94) | 1.637 (66) | 0.106 | -0.41, 4.17 |  |
| Child disclosure | 43 | 12.93 (3.09) |  | 24 | 11.80 (4.03) | 1.297 (66) | 0.199 | -0.61, 2.87 |  |
| Child secrecy | 43 | 6.02 (2.67) |  | 23 | 5.72 (2.85) | 0.441 (66) | 0.661 | -1.07, 1.68 |  |
| Parental control | 43 | 14.63 (4.77) |  | 24 | 12.52 (3.86) | 1.880 (65) | 0.065 | -0.13, 4.35 |  |
| FOH | 40 | 8.58 (4.04) |  | 24 | 7.71 (2.95) | 0.928 (65) | 0.357 | -1.00, 2.75 |  |
| Family cohesion | 41 | 18.18 (4.75) |  | 22 | 18.45 (4.70) | -0.226 (65) | 0.822 | -2.68, 2.13 |  |
| Family conflict | 42 | 10.86 (2.78) |  | 23 | 9.79 (2.84) | 1.496 (65) | 0.140 | -0.358, 2.50 |  |
| Note: FOH Feelings of being overly controlled | | | | | | | | |  |
